# AQP4 Antibody Assay Sensitivity Comparison in the Era of the 2015 Diagnostic Criteria for NMOSD

**DOI:** 10.3389/fneur.2019.01028

**Published:** 2019-10-04

**Authors:** Kerri Prain, Mark Woodhall, Angela Vincent, Sudarshini Ramanathan, Michael H. Barnett, Christine S. Bundell, John D. E. Parratt, Roger A. Silvestrini, Wajih Bukhari, The Australian and New Zealand NMO Collaboration, Fabienne Brilot, Patrick Waters, Simon A. Broadley

**Affiliations:** ^1^Pathology Queensland Central Laboratory, Division of Immunology, Royal Brisbane and Women's Hospital, Herston, QLD, Australia; ^2^Oxford Autoimmune Neurology Group, Nuffield Department of Clinical Neurosciences, University of Oxford, Oxford, United Kingdom; ^3^Brain Autoimmunity Group, Kids Neuroscience Centre, Kids Research at the Children's Hospital, Westmead, NSW, Australia; ^4^Brain and Mind Centre, University of Sydney, Camperdown, NSW, Australia; ^5^School of Biomedical Science, Medicine, University of Western Australia, Nedlands, WA, Australia; ^6^PathWest Laboratory Medicine, Department of Immunology, QEII Medical Centre, Nedlands, WA, Australia; ^7^Department of Neurology, Royal North Shore Hospital, Sydney, NSW, Australia; ^8^School of Medicine, Gold Coast Campus, Griffith University, Southport, QLD, Australia; ^9^Department of Neurology, Gold Coast University Hospital, Southport, QLD, Australia

**Keywords:** neuromyelitis optica, autoantibody, aquaporin, myelin oligodendrocyte glycoprotein, astrocytopathy, demyelination

## Abstract

We have compared five different assays for antibodies to aquaporin-4 in 181 cases of suspected Neuromyelitis optica spectrum disorders (NMOSD) and 253 controls to assess their relative utility. As part of a clinically-based survey of NMOSD in Australia and New Zealand, cases of suspected NMOSD were referred from 23 centers. Clinical details and magnetic imaging were reviewed and used to apply the 2015 IPND diagnostic criteria. In addition, 101 age- and sex-matched patients with multiple sclerosis were referred. Other inflammatory disease (*n* = 49) and healthy controls (*n* = 103) were also recruited. Samples from all participants were tested using tissue-based indirect immunofluorescence assays and a subset were tested using four additional ELISA and cell-based assays. Antibodies to myelin oligodendrocyte glycoprotein (MOG) were also assayed. All aquaporin-4 antibody assays proved to be highly specific. Sensitivities ranged from 60 to 94%, with cell-based assays having the highest sensitivity. Antibodies to MOG were detected in 8/79 (10%) of the residual suspected cases of NMOSD. Under the 2015 IPND diagnostic criteria for NMOSD, cell-based assays for aquaporin-4 are sensitive and highly specific, performing better than tissue-based and ELISA assays. A fixed cell-based assay showed near-identical results to a live-cell based assay. Antibodies to MOG account for only a small number of suspected cases.

## Introduction

Neuromyelitis optica spectrum disorders (NMOSD) (1) encapsulate a variety of defined neurological clinical presentations associated with autoantibodies to aquaporin-4 (AQP4) (2). Detection of antibodies to AQP4 is of immense value in the accurate diagnosis and management of NMOSD, which represent about 1% of central nervous system (CNS) inflammatory disease (3). The current diagnostic criteria for NMOSD permit the inclusion of AQP4 antibody negative cases, but this requires additional radiological criteria (1).

Myelin oligodendrocyte glycoprotein (MOG) antibody-related demyelinating disease is emerging as another antibody mediated inflammatory disorder of the CNS which shares some overlapping features with NMOSD (4). In particular, a predilection for lesions of the optic nerve and spinal cord is seen in both conditions (5, 6). However, there are some very clear clinical distinctions between the two disorders. MOG antibody-related demyelinating disease accounts for up to one third of cases of pediatric demyelinating disease, often presenting with acute disseminated encephalomyelitis, a clinical picture that is rare in NMOSD (7). In addition, the distribution of the spinal cord lesions is subtly different with lesions of the high cervical spine (C1/2) being seen in NMOSD and lesions extending all the way to the conus being seen in MOG antibody-related demyelinating disease (8).

We recently performed a nationwide prevalence survey of NMOSD across Australia and New Zealand (9). We have compared the relative utility of a variety of AQP4 antibody assays and studied the prevalence of positivity for MOG antibodies in this population, with the aim of guiding best laboratory practice and interpretation of results for clinicians.

## Methods

### Case Ascertainment

Possible cases of NMOSD were identified through a network of 23 neurology clinics specializing in demyelinating diseases of the CNS (ICD-10 G35-G37) across Australia and New Zealand. These centers match the population distribution of both countries. Participating centers referred cases to the coordinating center in Queensland if they had features suggestive of NMOSD as previously described (9). Cases were excluded if no serum sample was supplied and results of prior AQP4 antibody testing were not available, insufficient clinical data to make a diagnosis were supplied or if an alternate diagnosis became apparent. All subjects provided written informed consent. Institutional human research ethics committee approval was obtained for all participating sites. The period of data collection was from 1 January 2011 to 31 December 2013. The 2015 International Panel for NMO Diagnosis (IPND) diagnostic criteria for NMOSD (ICD-10 G36) were applied retrospectively.

Referring neurologists were also requested to recruit age- and sex-matched patients with multiple sclerosis, who did not have any of the features suggestive of NMOSD. Additional controls consisted of patients with other inflammatory diseases (infectious and rheumatological) and healthy blood donors. The other inflammatory diseases included infections (varicella, systemic CMV, infectious mononucleosis), Sjögren's syndrome and systemic lupus erythematosus. All participants gave written, informed consent to participation in this study and the study protocol was approved by the Human Research Ethics Committee at all participating sites.

Demographic details (age and gender) together with clinical details sufficient to confirm a diagnosis of NMOSD or MS were collected, including relapse history and MR imaging as previously described (9). Cases were then defined as “NMOSD” (meeting seropositive or seronegative 2015 IPND criteria) (1), “suspected NMOSD” (cases having features suggestive of NMOSD but not meeting 2015 IPND criteria), or “MS” (meeting 2010 McDonald criteria with no features suggestive of NMOSD) (10). The remaining control groups were other inflammatory disease and healthy blood donors.

### Antibody Assays

**Figure 1:**
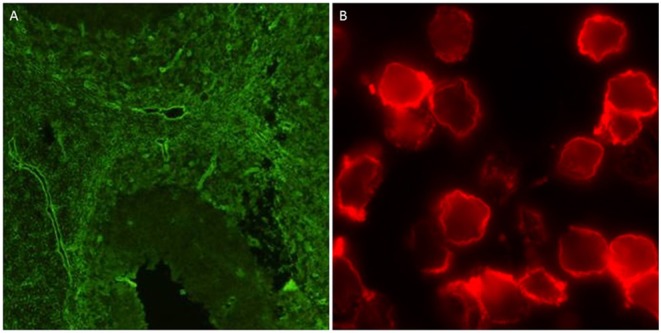
Positive outputs for AQP4 antibodies using tissue-based indirect immunofluorescence on mouse cerebellum **(A)**, and live cell-based assay **(B)**.

Any prior AQP4 antibody testing results were collected using a standard questionnaire in all cases. Serum samples were obtained and tested for AQP4 antibodies using indirect immunofluorescence staining techniques on mouse, rat, or monkey brain tissue and rat or mouse kidney sections at one of four testing sites (see Supplementary Table 1 for details). A subset of samples was also tested using an ELISA kit (RSRTM, UK), as well as two fixed cell-based slide kits from Euroimmun® and a live cell based assay (11). The two slides each consisted of two chips of HEK cells transfected with M1 and M23 isoforms of AQP4 in one and M23 AQP4 and MOG in the other (Euroimmun®, Germany). The tissue-based indirect immunofluorescence testing was undertaken in 4 centers across Australia. The ELISA, and fixed-cell based assays were performed by the Autoimmunity section of the Division of Immunology, Pathology Queensland Central Laboratory, Brisbane, Australia, as per the manufacturer's instructions. The live cell-based assay was performed in the Nuffield Department of Clinical Neurosciences, Oxford, UK, as previously described (12). All assays were performed by researchers blinded to the final diagnostic status of the cases and results from Brisbane and Oxford were collated by a blinded third party based in Cambridge, UK, who then distributed the final combined results to all parties. The typical outputs of the tissue-based and live cell-based assays are shown in Figure 1. MOG antibodies were detected using three different assays: a commercial fixed cell-based assay (Euroimmun®, Germany), a live cell-based assay, and a live cell-based fluorescence activated cell sorting (FACS) assay. The fixed cell-based assay was performed as per the manufacturer's instructions. The live cell-based assay was performed in the Nuffield Department of Clinical Neurosciences, Oxford, UK, as previously described (13) and the FACS assay was performed at the Westmead Immunology Laboratory, Sydney, Australia as previously described (14). Seropositivity for AQP4 or MOG antibodies was defined as either a positive result on any of the tissue-based indirect immunofluorescent assays or a positive result on at least 2 cell-based assays (including repeated FACS assay for MOG antibodies).

### Statistics

Results are presented as n/N (%) of positive or negative antibody assays in cases and controls. Non-parametric statistics were used to assess differences in the demographic distribution of cases and controls. The optimal cut-off for the ELISA antibody level was assessed using receiver operator characteristic (ROC) curve analysis. Sensitivity and specificity with 95% confidence intervals (CI) were used to assess utility of the assays. Degree of agreement between the assays was assessed using Cohen's kappa coefficient. All statistical analysis was performed using SPSS® v24 (IBM®, US).

## Results

In total, 189 cases of suspected NMOSD were referred. Of these 8/189 (4%) were excluded due to lack of an available serum sample. Of 181 suspected NMOSD cases, 80 met the 2015 IPND diagnostic criteria for NMOSD. Of these, 73/80 (91%) were seropositive for AQP4 antibodies and 7/80 (9%) were seronegative, leaving 101 suspected NMOSD cases. Not all of the seronegative NMOSD cases were tested with all assays. There were 108 cases of MS referred of which 7/108 (6%) had no serum available, leaving 101 included MS controls. Serum was available for 49 inflammatory disease and 103 blood donor controls. The inflammatory disease controls included the following: systemic lupus erythematosus (15), Sjögren's syndrome (8), cytomegalovirus infection (9), Epstein-Barr virus infection (7), and varicella zoster infection (6). The demographic details for cases and controls are given in Table 1. There were no statistically significant differences in gender (*X*^2^ = 0.503, *p* = 0.478) or age distribution (Mann-Whitney *U p* = 0.145) between NMOSD cases and MS controls, indicating that our age- and sex-matching strategy had been effective. No data were available for the blood donor controls as these samples were provided anonymously as required by Australian Red Cross. The inflammatory disease controls were older, but when combined with the MS controls were not significantly different to NMOSD cases. The proportion of females in inflammatory disease controls (61%) compared with NMOSD cases (89%) was significantly lower (*X*^2^ = 13.548, *p* < 0.001). When MS and inflammatory disease controls were combined the proportion of females increased (77%), but remained significantly different (*X*^2^ = 4.474, *p* = 0.034).

**Table 1:** Demographic details of cases and controls.

**Group tested**	***N***	**Gender, female n/N (%)**	**Age, years median (range)**
**CASES**			
NMOSD	80	71/80 (89)	47 (19-85)
Suspected NMOSD	101	68/101 (67)^*^	40 (15 – 72)^*^
**CONTROLS**			
Multiple sclerosis	101	86/101 (85)	46 (16 – 73)
Inflammatory disease	49	30/49 (61)^*^	59 (21 – 97)^*^
Blood donors	103	N/A	N/A
Overall	253	116/150 (77)^*^	49.5 (16 – 97)

* *Statistically significantly different from NMOSD cases (p < 0.05). NMOSD, neuromyelitis optica spectrum disorders*.

**Figure 2:**
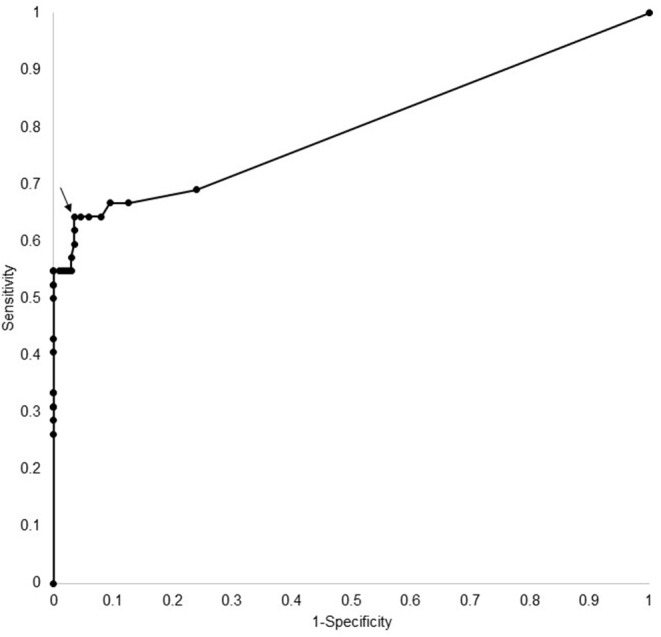
ROC curve analysis for most appropriate cut off (arrow) for ELISA AQP4 assay.

ROC curve analysis (see Figure 2) of the ELISA test kit results showed an optimal cut-off of equal to or >10 (arbitrary units), which had a sensitivity of 60% (95% CI 45–98%) and specificity of 97% (95% CI 93–98%). This level was used to determine positivity on the ELISA assay.

Tissue-based indirect immunofluorescence testing for AQP4 antibodies was performed in 424/434 (98%) of cases and controls. A cell-based AQP4 assay was performed in 307/434 (71%) of cases and controls. The sensitivity for various assays in NMOSD and suspected NMOSD together with their specificity in the various control groups and overall controls is given in Table 2. The results of the Euroimmun® M1 and M23 biochips on a shared slide proved to be identical and so these results have been considered together. The most sensitive assays were the fixed and live cell-based assays, which gave very similar results (see Table 2 and Supplementary Table 2). The overall sensitivity of the live cell-based assay was 92% (95% CI 78–97%) and specificity was 100% (95% CI 98–100%). Whilst less sensitive (78% [95% CI 69–87%]), the tissue-based indirect immunofluorescence assay also proved to be very specific (99.6% [95% CI 98–100%]). The ELISA test was positive in 6 inflammatory disease controls, but none of the blood donor or MS controls. The ELISA assay proved to be the least sensitive (60% [95%CI 45–98%]) and least specific (97% [95% CI 93–98%]).

**Table 2:** Sensitivity and specificity of autoantibody assays.

**Group tested**	***N***	**T-IIF**	**ELISA**	**EI-M1/M23**	**EI-CBA**	**Ox-CBA**	**MOG**
**CASE SENSITIVITY—n** **+ve/*****N*** **(%)**							
NMOSD [95% CI for sensitivity]	80	62/78 (78) [69–87]	25/42 (60) [45–73]	38/42 (90) [78–96]	34/36 (94) [82–99]	33/36 (92) [78–97]	0/48 (0) [0–7]
Suspected NMOSD	101						8/79 (10)
**CONTROL SPECIFICITY—n –ve/*****N*** **(%)**							
Suspected NMOSD	101	99/99 (100)	62/64 (97)	61/64 (95)	42/43 (98)	49/49 (100)	
Multiple sclerosis	101	98/98 (100)	48/48 (100)	48/48 (100)	20/20 (100)	21/21 (100)	52/52 (100)
Inflammatory disease	49	49/49 (100)	43/49 (88)	49/49 (100)	49/49 (100)	49/49 (100)	48/49 (98)
Blood donors	103	99/100 (99)	102/103 (99)	103/103 (100)	103/103 (100)	82/82 (100)	89/90 (99)
Overall [95% CI for specificity]	354	346/346 (99.7) [98–100]	255/264 (97) [94–98]	242/245 (99) [97–100]	214/215 (99.5) [97–100]	201/201 (100) [98–100]	189/191 (99) [96–100]

*T-IIF, tissue-based indirect immunofluorescence; ELISA, enzyme linked immunosorbent assay; EI M1/M23, Euroummun® M1/M23 biochip slide; EI-CBA, Euroimmun® AQP4 fixed cell-based assay; Ox-CBA, Oxford AQP4 live cell-based assay; MOG, myelin oligodendrocyte glycoprotein antibody assay; NMOSD, neuromyelitis optica spectrum disorders*.

The degree of concordance between assays was generally high, and particularly so for the cell-based assays, as shown in Table 3. In the suspected NMOSD cases, there were 5 cases who were positive on the Euroimmun® M1/M23 assay or the ELISA assay alone. As these cases were negative on all other cell-based assays they were not included in the NMOSD cases and remained as suspected NMOSD. Inclusion of the suspected NMOSD cases as controls for the calculation of specificity did not significantly change the results.

**Table 3:** Concordance and agreement for AQP4 antibody assays.

**Assay**	**T-IIF**	**ELISA**	**EI M1/M23**	**EI AQP4**
**ELISA**	121/141 (86)			
	**0.556**	n/a		
	*<0.001*			
**EI M1/M23**	131/141 (93)	121/141 (86)		
	**0.790**	**0.605**	n/a	
	*<0.001*	*<0.001*		
**EI AQP4**	132/141 (94)	122/141 (87)	136/141 (96)	
	**0.808**	**0.620**	**0.904**	n/a
	*<0.001*	*<0.001*	*<0.001*	
**Ox AQP4**	134/141 (95)	122/141 (87)	136/141 (96)	139/141 (99)
	**0.847**	**0.612**	**0.902**	**0.960**
	*<0.001*	*<0.001*	*<0.001*	*<0.001*

*All data presented as: Concordance n/N (%); bold values represent the Cohen's kappa coefficient; italic value represent the P-value; n/a, not applicable*.

*T-IIF, tissue-based indirect immunofluorescence; ELISA, enzyme linked immunosorbent assay; EI M1/M23, Euroummun® M1/M23 biochip slide; EI-CBA, Euroimmun® AQP4 fixed cell-based assay; Ox-CBA, Oxford AQP4 live cell-based assay*.

**Figure 3:**
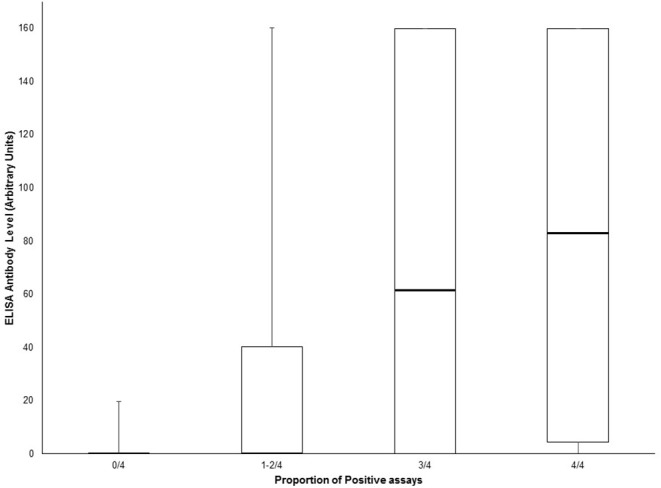
Box and whisker plot of ELISA antibody levels according to the proportion of positive AQP4 assays (tissue-based indirect immunofluorescence, Euroimmun® M1/M23 biochip slide, Euroimmun® AQP4/MOG biochip slide and Oxford live cell-based assay). Central bar shows the median, boxes represent interquartile range, and whiskers indicate range.

Amongst suspected NMOSD cases, 8 were positive for MOG antibodies. One of these was also positive for both the AQP4 and MOG biochips on the same fixed cell-based assay. This case was negative for all other cell-based assays for AQP4 antibodies and was confirmed as positive for MOG antibodies by FACS assay and so was not considered to be a case of NMOSD, but rather as a case of MOG antibody-related demyelinating disease. Thus, we did not identify any AQP4 and MOG antibody double positive cases. One MOG antibody positive case met the clinical/MRI 2015 IPND criteria for a diagnosis of NMOSD, but was considered as a MOG antibody-related demyelinating disease case. When the sensitivity and specificity analysis was restricted to cases with testing available for all assays (AQP4 and MOG) results were not significantly different (Supplementary Tables 2, 3). We observed a clear correlation between the number of positive tests (tissue and cell-based assays) and the ELISA antibody level (Figure 3). However, antibody levels >100 were seen in a few samples with only one positive result on the other assays.

## Discussion

We have conducted a rater-blinded comparison of 5 different assays for antibodies to AQP4 in a population of cases with suspected NMOSD and a variety of controls. Consistent with previous studies (11, 12, 16, 17) we have found that the sensitivity of cell-based assays, both fixed and live cell-based assays, was higher (90–94%) than for either an ELISA assay (60%) or tissue indirect immunofluorescence (78%). The sensitivity of cell-based assays was at the higher end of previously reported data for studies using the 2006 Wingerchuk or earlier diagnostic criteria for NMOSD in adult, Caucasian populations (64–98%) (11, 12, 15, 18–20). This likely reflects the stricter radiological requirements of the 2015 IPND criteria. In addition to having typical presenting attacks, cases must also fulfill additional MRI criteria for the commoner presenting lesions (e.g., longitudinally extensive spinal cord lesion, long optic nerve lesion or area postrema lesion on imaging). All assays proved to be highly specific (≥97%) with the Euroimmun® fixed cell-based AQP4 and live cell based assays showing 100% specificity. False positives were more common amongst cases with MS and other inflammatory diseases with the ELISA assay having the highest false positive rate. This finding has also been noted previously (17). The concordance between assays, particularly for the cell-based assays was high.

The status of cases with positive results for one cell-based assay in the remaining suspected NMOSD cases remains uncertain. This may reflect greater sensitivity in “true positives.” However, they may also represent false positives. Currently, there is no means to determine the true status of these cases although repeat testing over time may prove useful. The fact that the number of positive tests correlates well with the ELISA antibody level suggests that false negative results may occur when antibody levels are low, reflecting a sensitivity issue. However, the possibility of this being due to lower specificity of these assays cannot be discounted.

Amongst suspected NMOSD cases who were seronegative for AQP4 antibodies and did not meet the 2015 IPND diagnostic criteria for NMOSD 8/79 (10% [95% CI 5–19%]) were positive for MOG antibodies. This is again consistent with previous studies that have shown positivity rates for MOG antibodies in this population of 8–32% (17, 19, 21, 22). Specificity for MOG antibodies was 190/191 (99% [95% CI 96–100%]). No cases were positive for both AQP4 (on more than one assay) and MOG antibodies.

One advantage of the present study was that all cases were identified clinically by clinicians experienced in diagnosing inflammatory disease of the CNS and not based upon the results of laboratory testing, which introduces an inherent bias and the potential for low pre-test probability. The fact that not all cases were assessed using all assays is a weakness in this study, but when the analysis was restricted to only cases tested for all AQP4 antibody assays the results were not significantly different. The finding of identical results for the M1 and M23 AQP4 antibody assays is contrary to prior studies which have indicated a higher sensitivity for the M23 isoform (18). However, another recent study found the same result (23). The lack of clinical inclusion criteria for rarer presentations which had not been defined at the time of this study (e.g., area postrema lesion) is a further weakness of this study. Cases with these features were included and the numbers of missed cases is likely to have been small. However, depending on the relative frequency of positive AQP4 antibodies in these cases this could have had an impact on the reported sensitivity. There is no data to suggest that the rate of seropositivity in these cases would be different.

We have confirmed the high sensitivity and specificity for a wide range of AQP4 antibody assays in identifying NMOSD. The high sensitivity is to be expected, because of the inclusion of positive AQP4 antibodies as a part of the diagnostic criteria in the presence of a single characteristic presentation (1). The higher sensitivity of cell-based assays makes these preferable over other AQP4 assays in the identification of NMOSD. The fact that more than half of all suspected NMOSD cases are negative for both AQP4 and MOG antibodies remains a diagnostic dilemma. The issue of whether these cases are false negatives on the available assays or represent phenocopies of NMOSD remains unresolved. It is possible that yet more antibodies remain to be identified in this patient population or that a T-cell mediated process more akin to that hypothesized for MS pathology may be responsible for these cases (24). The high specificity of both AQP4 and MOG antibody assays means that in clinical practice, where there is a characteristic clinical presentation, a positive antibody result can be taken as being indicative of NMOSD or MOG antibody-related demyelinating disease respectively. Caution should be applied in the setting of concurrent inflammatory diseases, due to potential false positive results. The recent 2015 IPND criteria identify a closely defined group of NMOSD cases suitable for research purposes, but leaves a wider group of cases with a similar phenotype unclassified.

## Data Availability Statement

De-identified, individual level data for cases where all assays were performed is provided as Supplementary Table 2.

## Ethics Statement

The studies involving human participants were reviewed and approved by Griffith University HREC. The patients/participants provided their written informed consent to participate in this study.

## Author Contributions

DA, MHB, SBh, SBl, MB, KB, BB, SAB, WB, CB, HB, WC, CC, LC, AC, RD, CD, KD, DG, SHa, RH, AH, SHe, SHo, AGK, TK, JK, CK, CL, RM, MM, DM, PM, CO'G, JDEP, MP, JP, JDP, KP, SWR, SS, JL-S, CS, RS, MS, JSp, JSt, IS, BT, AV, SV, MWa, PW, EW, RJW, and RCW conceived and designed the study. DA, MHB, SBh, SBl, MB, KB, BB, FB, SAB, WJB, WB, CB, HB, WC, CC, LC, AC, RD, CD, DF, DG, SH, RH, AH, SHe, SHo, AK, TK, JK, CK, AJK, M-WL, CL, RM, MM, DM, PM, CO'G, JDEP, MF-P, MP, JP, JDP, KP, SR, SWR, SS, JL-S, CS, RS, MS, JSp, JSt, IS, BT, SV, MWa, PW, EW, RJW, RCW, MWo, and EY played a major role in collecting data. FB, SAB, WB, KD, KP, SR, JSt, and PW conducted the analyses. KP, WB, and SAB prepared the initial draft. MHB, BB, FB, SAB, CB, HB, WC, LC, RD, KD, DG, SHo, AGK, TK, JK, RM, MM, DFM, PM, CO'G, JDEP, MF-P, MP, JP, JDP, SR, SWR, JL-S, CS, RS, MS, JSp, JSt, IS, BT, AV, SV, PW, EW, and MWo contributed to revisions. All authors approved the final draft.

### Conflict of Interest

MHB has received honoraria for participation in advisory boards and travel sponsorship from Novartis, BioCSL, Genzyme, and Biogen Idec. MB has received travel sponsorship and honoraria from Sanofi-Genzyme, Teva, Novartis, Biogen Idec, and Roche. BB has received honoraria as a board member for GlaxoSmithKline, Biogen Idec, ViiV Healthcare, and Merck Serono, has received speaker honoraria from ViiV Healthcare, Boehringer Ingelheim, Abbott, Abbvie, and Biogen Idec; has received travel sponsorship from Abbott and Viiv Healthcare, and has received research support funding from EI Lilly, GlaxoSmithKline, ViiV Healthcare and Merck Serono. SAB has received honoraria for attendance at advisory boards and travel sponsorship from Bayer-Scherring, Biogen-Idec, Merck-Serono, Novartis, and Sanofi-Genzyme, has received speakers honoraria from Biogen-Idec and Genzyme, is an investigator in clinical trials sponsored by Biogen Idec, Novartis, and Genzyme, and was the recipient of an unencumbered research grant from Biogen-Idec. HB has received honoraria for serving on scientific advisory boards for Biogen Idec, Novartis, and Sanofi-Genzyme, has received conference travel sponsorship from Novartis and Biogen Idec, has received honoraria for speaking and acting as Chair at educational events organized by Novartis, Biogen Idec, Medscape, and Merck Serono, serves on steering committees for trials conducted by Biogen Idec and Novartis, is chair (honorary) of the MSBase Foundation, which has received research support from Merck Serono, Novartis, Biogen Idec, Genzyme Sanofi, and CSL Biopharma, and has received research support form Merck Serono. WC has been the recipient of travel sponsorship from, and provided advice to Bayer Schering Pharma, Biogen-Idec, Novartis, Genzyme, Sanofi-Aventis, BioCSL, and Merck-Serono. RD has received research funding from the National Health and Medical Research Council, MS Research Australia, Star Scientific Foundation, Pfizer Neuroscience, Tourette Syndrome Association, University of Sydney, and the Petre Foundation and has received honoraria from Biogen-Idec and Bristol-Myers Squibb as an invited speaker. MF-P has received travel sponsorship from Biogen-Idec and Merck Serono. RH has received honoraria, educational support and clinic funding from Novartis, Biogen Idec, Genzyme and BioCSL. AGK has received scientific consulting fees and/or lecture honoraria from Bayer, BioCSL, Biogen-Idec, Genzyme, Merck, Novartis, Sanofi-Aventis, and Teva. TK has received travel sponsorship from Novartis, BioCSL, Novartis, Merck Serono, and Biogen Idec, has received speaker honoraria from Biogen Idec, BioCSL, Merck Serono, Teva, Genzyme, and Novartis, has received research support from Biogen Idec, Genzyme, GlaxoSmithKline, Bayer-Schering, and Merck Serono, and has received scientific consulting fees from GlaxoSmithKline China, Biogen-Idec and Novartis. JK has received remuneration for advisory board activities and presentations from Bayer Healthcare, Biogen Idec, BioCSL, Genzyme and Novartis. CK has received travel support, honoraria and advisory board payments from Biogen Idec, Bayer, Genzyme, Novartis, and Serono. JL-S has received unencumbered funding as well as honoraria for presentations and membership on advisory boards from Sanofi Aventis, Biogen Idec, Bayer Health Care, CSL, Genzyme, Merck Serono, Novartis Australia, and Teva. RM has received honoraria for attendance at advisory boards and travel sponsorship from Bayer-Scherring, Biogen-Idec, CSL, Merck-Serono, Novartis, and Sanofi-Genzyme. MM has received travel sponsorship, honoraria, trial payments, research and clinical support from Bayer Schering, Biogen Idec, BioCSL, Genzyme, Novartis, and Sanofi Aventis Genzyme. DM has received honoraria for attendance at advisory boards from Biogen-Idec and Novartis, and travel sponsorship from Bayer-Scherring, Biogen-Idec, and Sanofi-Genzyme. PM has received honoraria or travel sponsorship from Novatis, Sanofi-Aventis, and Biogen Idec. JAP has received travel sponsorship, honoraria for presentations and membership on advisory boards from Biogen Idec and Novartis and Sanofi Aventis. JDP has received honoraria for seminars or advisory boards from Teva, Biogen, Sanofi-Genzyme, Novartis, Merck, Bayer, and research grants or fellowships from Merck, Novartis, Bayer, Biogen, Sanofi-Genzyme, and Teva. SR has received travel sponsorship, honoraria, trial payments, research and clinical support from Aspreva, Baxter, Bayer Schering, Biogen Idec, BioCSL, Genzyme, Novartis, Sanofi Aventis Genzyme, and Servier, and is a director of Medical Safety Systems Pty Ltd. CS has received travel sponsorship from Biogen Idec, Novartis, and Bayer-Schering. IS has received remuneration for Advisory Board activities from Biogen, CSL, and Bayer Schering and educational activities with Biogen, CSL, and travel sponsorship from Biogen, Novartis, and Bayer Schering. MS has received research support from Novartis, Biogen Idec, and BioCSL. JSp has received honoraria for lectures and participation in advisory boards, and travel sponsorship from Novartis, BioCSL, Genzyme, and Biogen Idec. BT has received travel sponsorship from Novartis and Bayer Schering. AV and the University of Oxford hold patents and receive royalties for antibody testing. PW and the University of Oxford hold patents for antibody assays and have received royalties, has received speaker honoraria from Biogen Idec and Euroimmun® AG, and travel grants from the Guthy-Jackson Charitable Foundation. EW has received honoraria for participation in advisory boards from Biogen-Idec and Novartis, travel sponsorship from Biogen-Idec, Bayer-Schering and Teva and is an investigator in clinical trials funded by Biogen-Idec and Teva. MP has received consulting fees and research funding from Atara Biotherapeutics. The remaining authors declare that the research was conducted in the absence of any commercial or financial relationships that could be construed as a potential conflict of interest.
