# Supplementary material for: AQP4 Antibody Assay Sensitivity Comparison in the Era of the 2015 Diagnostic Criteria for NMOSD
Source: Front Neurol. 2019 Oct 4;10:1028. doi: 10.3389/fneur.2019.01028 (PMC6787171; doi:10.3389/fneur.2019.01028)
Supplement: Supplementary file 1 [file Table_1.docx]

## Supplementary Table 1. Summary of tissue-based indirect immunofluorescence protocols

| Laboratory | Brisbane | University of Sydney | Westmead | Perth |
| --- | --- | --- | --- | --- |
| Substrate | In-house composite of unfixed mouse cerebellum, midbrain, stomach and renal papillae | In-house composite of fixed (cold acetone) rat cerebellum, cerebral cortex and (whole) kidney | Composite of monkey cerebellum and midbrain, and rodent stomach. (Binding Site^®^ or IMMCO^®^) and in-house mouse (whole) kidney | Composite of monkey cerebellum and cerebrum, and mouse stomach (INOVA^®^) and in-house fixed (acetone) rat (whole) kidney |
| Pre-treatment | Nil | Liver powder for 60 mins (rotator in cold room) with 1 part liver powder to 3 parts diluted serum | Only if ANA or other autoantibodies of high titre interfere with the reading, test is repeated after pre-treatment of serum with rodent liver powder | Nil |
| Screening Serum Dilution | 1/40 | 1/60 (1/80 effectively due to above) | Screening dilution: 1/10 | Screening dilution: 1/10 |
| Titration Steps | 1/160, 1/640 | 1/120, 1/180, 1/240 etc. | 1/40, 1/160, 1/640 | 1/40, 1/160, 1/640 |
| Serum Incubation Time | 30 minutes (on CARIS™ Automated Slide Processer) or 20 minutes (on Bench) room temperature | 60 minutes (on Bench)  Room temperature | 45 minutes (on Bench)  Room temperature | 30 minutes  Room temperature |
| Washes | Bench: 3 x 4 mins (PBS, post serum) + 3 x 4 mins (PBS, post conjugate)  CARIS^TM^: constant stream (PBS) for 3-5 seconds post serum & post conjugate | Bench: 3 x 5 mins (PBS, post serum) + 3 x 5 mins (PBS, post conjugate) | Bench: 15 mins (PBS, post serum) + 15 mins (PBS, post conjugate) | Bench: 1 rinse followed by 1 x 10 min wash (PBS, post serum) + 3 x 10 minutes wash (PBS, post conjugate)  CARIS^TM^: 5 washes per well post serum and post conjugate |
| Conjugate | FITC conjugated goat anti-human IgG (heavy chain only), diluted 1/200 (Southern Biotech^®^) | Alexa^®^ Fluor 488 (or FITC) – conjugated goat anti-human IgG (heavy and light chain), diluted 1/1000 dilution (Invitrogen^®^) | FITC conjugated rabbit anti-human IgG (heavy chain only), 1/100 (Dako^®^) | INOVA^®^ Slide: FITC conjugated monkey-absorbed anti-human IgG, heavy chain only (INOVA^®^)  Kidney Slide: FITC conjugated sheep or goat anti-human IgG (heavy chain only) (Southern Biotech^®^ or Millipore^®^) |
| Mounting Medium | PBS / 30% Glyceryl, pH 8.0 (in-house) | ProLong^®^ Gold antifade reagent (Invitrogen^®^) | IMMCO^®^ Mountant | Immunoconcepts^®^ |
| Microscope | Olympus^®^ BX41 or BX60 x200 | Carl Zeiss^®^ Axio Imager M1 x200 | Olympus^®^ BX41 x200 | QI^®^ Lamp x160 |
| Colocalization Step | Nil | With monoclonal anti-AQP4 (Alexa^®^ Fluor 610) | Nil | Nil |

# ANA = anti-nuclear antigen; FITC = fluorescein isothiocyanate; IgG = imnnunoglobulin G

**Supplementary Table 2. AQP4 antibody assay results for cases where all tests were performed**

**Supplementary Table 3 – Sensitivity and specificity of assays where complete data for all assays available**

| Group tested | N | T-IIF | ELISA | EI M1/M23 | EI AQP4 | Ox AQP4 | MOG |
| --- | --- | --- | --- | --- | --- | --- | --- |
| Case Sensitivity – n +ve/N (%) | | |  |  |  |  |  |
| NMOSD  [95% CI for Sensitivity] | 80 | 25/34 (74)  [57 – 85] | 23/34 (68)  [51 – 81] | 32/34 (94)  [81 – 98] | 33/34 (97)  [85 – 100] | 32/34 (94)  [81 – 98] | 0/34(0)  [0 – 10] |
| Suspected NMOSD | 101 |  |  |  |  |  | 5/41 (12) |
| Control Specificity – n -ve/N (%) | |  |  |  |  |  |  |
| Suspected NMOSD | 101 | 41/41 (0) | 39/41 (5) | 39/41 (5) | 40/41 (2) | 41/41 (0) |  |
| Multiple Sclerosis | 101 | 17/17 (100) | 17/17 (100) | 16/17 (94) | 17/17 (100) | 17/17 (100) | 17/17 (100) |
| Inflammatory Disease | 49 | 49/49 (100) | 43/49 (88) | 49/49 (100) | 49/49 (100) | 49/49 (100) | 48/49 (98) |
| Blood Donors | 103 |  |  |  |  |  |  |
| Overall  [95% CI for Specificity] | 253 | 107/107 (100)  [97 – 100] | 99/107 (93)  [86 – 96] | 104/107 (97)  [92 – 99] | 106/107 (99)  [95 – 100] | 107/107 (100)  [97 – 100] | 65/66 (98)  [92 – 100] |

T-IIF = tissue-based indirect immunofluorescence; ELISA = enzyme linked immunosorbent assay; EI M1/M23 = Euroummun^®^ M1/M23 biochip slide; EI-CBA = Euroimmun^®^ AQP4 fixed cell-based assay; Ox-CBA = Oxford AQP4 live cell-based assay; MOG = myelin oligodendrocyte glycoprotein antibody assay; NMOSD = neuromyelitis optica spectrum disorders
