# Supplementary material for: AQP4 Antibody Assay Sensitivity Comparison in the Era of the 2015 Diagnostic Criteria for NMOSD
Source: Front Neurol. 2019 Oct 4;10:1028. doi: 10.3389/fneur.2019.01028 (PMC6787171; doi:10.3389/fneur.2019.01028)
Supplement: Supplementary file 2 [file Data_Sheet_1.docx]

**Appendix 1**

| Name | Location |
| --- | --- |
| Kerri M Prain | Pathology Queensland Central Laboratory, Brisbane, Australia |
| Mark Woodhall | Nuffield Department of Clinical Neurosciences, Oxford, UK |
| Angela Vincent | Nuffield Department of Clinical Neurosciences, Oxford, UK |
| Sudarchini Ramanathan | Institute for Neuroscience and Muscle Research, Sydney, Australia |
| Michael H Barnett | Brain and Mind Research Institute, Sydney, Australia |
| Christine S Bundell | PathWest Laboratory Medicine, Perth, Australia |
| John D E Parratt | Brain and Mind Research Institute, Sydney, Australia |
| Roger A Silvestrini | Institute for Neuroscience and Muscle Research, Sydney, Australia |
| Wajih Bukhari | Griffith University, Gold Coast, Australia |
| Fabienne Brilot | Institute for Neuroscience and Muscle Research, Sydney, Australia |
| Patrick Waters | Nuffield Department of Clinical Neurosciences, Oxford, UK |
| Simon A Broadley | Griffith University, Gold Coast, Australia |
| The ANZ NMO Collaboration | |
| Cullen O'Gorman | Griffith University, Gold Coast, Australia |
| Laura Clarke | Griffith University, Gold Coast, Australia |
| David Abernethy | Wellington Hospital, Wellington, NZ |
| Sandeep Bhuta | Griffith University, Gold Coast, Australia |
| Stefan Blum | Princess Alexandra Hospital, Brisbane, Australia |
| Mike Boggild | Townsville Hospital, Townsville, Australia |
| Karyn Boundy | Royal Adelaide Hospital, Adelaide, Australia |
| Bruce J Brew | Centre for Applied Medical Research, Sydney, Australia |
| Wallace J Brownlee | Auckland City Hospital, Auckland, NZ |
| Helmut Butzkueven | Monash University, Melbourne, Australia |
| William M Carroll | Perron Institute for Neurological and Translational Science, Perth, Australia |
| Celia Chen | Flinders Medical Centre, Adelaide, Australia |
| Alan Coulthard | University of Queensland, Brisbane, Australia |
| Russell C Dale | Children's Hopsital Westmead, Sydney, Australia |
| Chandi Das | Canberra Hospital, Canberra, Australia |
| Keith Dear | University of Adelaide, Adelaide, Australia |
| Marzena J Fabis-Pedrini | Perron Institute for Neurological and Translational Science, Perth, Australia |
| David Fulcher | Westmead Hosptial, Sydney, Australia |
| David Gillis | Pathology Queensland Central Laboratory, Brisbane, Australia |
| Simon Hawke | Royal Prince Alfred Hospital, Sydney, Australia |
| Robert Heard | Westmead Hosptial, Sydney, Australia |
| Andrew P D Henderson | Westmead Hosptial, Sydney, Australia |
| Saman Heshmat | Griffith University, Gold Coast, Australia |
| Suzanne Hodgkinson | Liverpool Hospital, Sydney, Australia |
| Sofia Jimenez Sanchez | Griffith University, Gold Coast, Australia |
| Trevor J Kilpatrick | Florey Institute of Neuroscience and Mental Health, Melbourne, Australia |
| John King | Royal Melbourne Hospital, Melbourne, Australia |
| Chris Kneebone | Royal Adelaide Hospital, Adelaide, Australia |
| Andrew J Kornberg | Royal Children's Hospital, Melbourne, Australia |
| Jeanette Lechner-Scott | Hunter Medical Research Institute, Newcastle, Australia |
| Ming-Wei Lin | Royal Prince Alfred Hospital, Sydney, Australia |
| Chistopher Lynch | University of Auckland, Auckland, NZ |
| Richard A L Macdonnell | Austin Health, Melbourne, Australia |
| Deborah F Mason | Chistchurch Hospital, Christchurch, NZ |
| Pamela A McCombe | Centre for Clinical Research, University of Queensland, Brisbane, Australia |
| Michael P Pender | University of Queensland, Brisbane, Australia |
| Jennifer Pereira | University of Auckland, Auckland, NZ |
| John D Pollard | Brain and Mind Research Institute, Sydney, Australia |
| Stephen W Reddell | Brain and Mind Research Institute, Sydney, Australia |
| Cameron Shaw | Deakin University, Geelong, Australia |
| Judith Spies | Brain and Mind Research Institute, Sydney, Australia |
| James Stankovich | Menzies Research Institute, Hobart, Australia |
| Ian Sutton | St Vincent's Hospital, Sydney, Australia |
| Steve Vucic | Westmead Hosptial, Sydney, Australia |
| Michael Walsh | Princess Alexandra Hospital, Brisbane, Australia |
| Richard C Wong | Pathology Queensland Central Laboratory, Brisbane, Australia |
| Eppie M Yiu | Royal Children's Hospital, Melbourne, Australia |
| Allan G Kermode | Institute for Immunology and Infectious Diseases, Perth, Australia |
| Mark P Marriott | Royal Melbourne Hospital, Melbourne, Australia |
| Mark Slee | Flinders Medical Centre, Adelaide, Australia |
| Bruce V Taylor | Menzies Research Institute, Hobart, Australia |
| Ernest Willoughby | University of Auckland, Auckland, NZ |
| Robert J Wilson | Pathology Queensland Central Laboratory, Brisbane, Australia |
